# Spin-Selective Transmission and Devisable Chirality in Two-Layer Metasurfaces

**DOI:** 10.1038/s41598-017-08527-4

**Published:** 2017-08-15

**Authors:** Zhancheng Li, Wenwei Liu, Hua Cheng, Shuqi Chen, Jianguo Tian

**Affiliations:** The Key Laboratory of Weak Light Nonlinear Photonics, Ministry of Education, School of Physics and TEDA Institute of Applied Physics, Nankai University, Tianjin, 300071 China

## Abstract

Chirality is a nearly ubiquitous natural phenomenon. Its minute presence in most naturally occurring materials makes it incredibly difficult to detect. Recent advances in metasurfaces indicate that they exhibit devisable chirality in novel forms; this finding offers an effective opening for studying chirality and its features in such nanostructures. These metasurfaces display vast possibilities for highly sensitive chirality discrimination in biological and chemical systems. Here, we show that two-layer metasurfaces based on twisted nanorods can generate giant spin-selective transmission and support engineered chirality in the near-infrared region. Two designed metasurfaces with opposite spin-selective transmission are proposed for treatment as enantiomers and can be used widely for spin selection and enhanced chiral sensing. Specifically, we demonstrate that the chirality in these proposed metasurfaces can be adjusted effectively by simply changing the orientation angle between the twisted nanorods. Our results offer simple and straightforward rules for chirality engineering in metasurfaces and suggest intriguing possibilities for the applications of such metasurfaces in spin optics and chiral sensing.

## Introduction

Chirality, a signature aspect of materials in the organic world, is defined as the quality of objects that lack mirror-image symmetry in three-dimensional space^1, 2^. Chirality exists in a wide array of naturally occurring molecules, such as carbohydrates, amino acids, and proteins. Thus, chirality attracts the attention of researchers investigating the fundamentals of both biology and chemistry^1–4^. Contemporary research defines circular dichroism (CD) spectra as the difference in absorbance for left-handed circular-polarized (LCP) and right-handed circular-polarized (RCP) waves; CD spectra serve as the main means for chirality detection and discrimination. LCP and RCP waves have opposite spin and appear in processes that display inherent chirality^5^. Consequently, circular-polarized waves with opposite spins (LCP or RCP) interact differently with chiral materials^6^. However, the chirality of natural molecules is quite subtle, and its detection and discrimination are difficult.

Over the past decade, advances in metamaterials and metasurfaces have provided extensive opportunities for developments in chirality studies and sensing^6–10^. Compared to naturally occurring molecules, these artificial nanostructures show significantly enhanced chiral optical responses that are crucial to the understanding of chirality and provide an effective way to improve conformational and structural analyses in both biology and chemistry^11–13^. Recently, chirality in three-dimensional space leading to CD has gathered significant scientific interest, as has two-dimensional chirality and extrinsic chirality^14–17^. Previous research projects investigating chiral optical responses of artificial nanostructures have sparked the development of chiral nanophotonics.

In contemporary research, chiral artificial nanostructures have played important roles in spin optics and polarization controlling because of their polarization-selective responses. Rodrigues *et al*. demonstrated a spin-selective enhancement of two-photon luminescence from quantum emitters based on a chiral-arc metamaterial^18^. Wang *et al*. demonstrated spin-selective metamirrors that enable selective, near-perfect reflection of designated circularly polarized waves without reversing their handedness and provide complete absorption of the other polarization states^19^. In addition, Kenney *et al*. demonstrated a Pancharatnam–Berry phase-induced spin-selective transmission in herringbone-dielectric metamaterials^20^. These demonstrated artificial nanostructures provide revolutionary insights into spin-selective interactions between optical systems and electromagnetic waves and offer wide-ranging possibilities for nanophotonics applications. Thus, providing simple and straightforward rules for designing and engineering chiral optical responses of artificial nanostructures is important. An effective approach to selecting the spin of transmission-mode incident waves without polarization conversion also is desirable. Moreover, achieving a simple means for the continuous engineering of chiral optical responses of artificial nanostructures plays an irreplaceable role in applications employing spin optics and chiral sensing.

Here, we propose using two-layer metasurfaces based on twisted nanorods to generate spin-selective transmissions and to achieve chirality engineering in the near-infrared region. The proposed metasurfaces exhibit *C*_4_-symmetry relative to the wave-transmission direction but lack any additional reflection symmetry. This design is based on the theoretical predictions using advanced Jones calculus analysis and then demonstrated using simulations. We designed two metasurfaces with opposite spin-selective transmission at 1650 nm with over 50% efficiency. These metasurfaces can be treated as enantiomers and used as spin selectors between 1640 nm to 1652 nm. Moreover, we demonstrate that the chirality of the proposed metasurfaces can be engineered easily by simply changing the orientation angle of the twisted nanorods.

### Theoretical Analysis

Materials can be classified based on their structural symmetry. Here, advanced Jones calculus is used to analyze the chirality and theoretical conditions of spin-selective transmission in periodic metasurfaces^21^. The complex amplitudes of circularly polarized incident and transmitted fields can be related by the complex Jones matrix **T**, where

1 $$(\begin{array}{c}{t}_{{\rm{LCP}}}\\ {t}_{{\rm{RCP}}}\end{array})=(\begin{array}{cc}{t}_{{\rm{LL}}} & {t}_{{\rm{LR}}}\\ {t}_{{\rm{RL}}} & {t}_{{\rm{RR}}}\end{array})(\begin{array}{c}{i}_{{\rm{LCP}}}\\ {i}_{{\rm{RCP}}}\end{array})={{\boldsymbol{{\rm T}}}}_{{\rm{circ}}}(\begin{array}{c}{i}_{{\rm{LCP}}}\\ {i}_{{\rm{RCP}}}\end{array}).$$

Here, the subscript “circ” of **T**_circ_ indicates that the Jones matrix lies in the circular base.

CD spectra always can characterize the chirality of structures. For structures with *C*_4_-symmetry respect to the *z* axis, the difference of the transmission intensity Δ*T* for RCP and LCP illumination is directly correlated to CD, so that

2 $${\rm{\Delta }}T={T}_{{\rm{LCP}}}-{T}_{{\rm{RCP}}},$$

where *T*_*i*_ = |*t*_*i*_|^2^ is the squared moduli of the transmitted electric field.

For the structures with *C*_4_-symmetry relative to the *z* axis but without any additional reflection symmetry, The **T** matrix in the linear base is

3 $${{\bf{T}}}_{{\rm{lin}}}=(\begin{array}{cc}{t}_{xx} & {t}_{xy}\\ -{t}_{xy} & {t}_{xx}\end{array}),$$

the **T**_circ_ can then be obtained by a change of the base vectors^21, 22^

4 $${{\bf{T}}}_{{\rm{circ}}}=(\begin{array}{cc}{t}_{{\rm{LL}}} & {t}_{{\rm{LR}}}\\ {t}_{{\rm{RL}}} & {t}_{{\rm{RR}}}\end{array})=\frac{1}{2}(\begin{array}{cc}{t}_{xx}+{t}_{yy}+i({t}_{xy}-{t}_{yx}) & {t}_{xx}-{t}_{yy}-i({t}_{xy}+{t}_{yx})\\ {t}_{xx}-{t}_{yy}+i({t}_{xy}+{t}_{yx}) & {t}_{xx}+{t}_{yy}-i({t}_{xy}-{t}_{yx})\end{array}),$$

then, the **T** matrix in the circular base is

5 $${{\bf{T}}}_{{\rm{circ}}}=(\begin{array}{cc}{t}_{{\rm{LL}}} & 0\\ 0 & {t}_{{\rm{RR}}}\end{array})=(\begin{array}{cc}{t}_{xx}+i{t}_{xy} & 0\\ 0 & {t}_{xx}-i{t}_{xy}\end{array}).$$

The difference of the transmission intensity Δ*T* is then given by

6 $${\rm{\Delta }}T={T}_{{\rm{L}}{\rm{C}}{\rm{P}}}-{T}_{{\rm{R}}{\rm{C}}{\rm{P}}}=-4|{t}_{xx}|\cdot |{t}_{xy}|\cdot \,\sin ({\phi }_{xy}-{\phi }_{xx}),$$

where *φ*_*ij*_ is the phase of the complex coefficients *t*_*ij*_. Thus, the chirality of this kind of structure is determined by the amplitude and phase difference of the linear-based complex coefficients *t*_*xx*_ and *t*_*xy*_.

Either *t*_LL_ or *t*_RR_ must be zero for a spin-selective transmission to be generated. Thus, the amplitudes of complex coefficients *t*_*xx*_ and *t*_*xy*_ must be equal while their phases must show a difference of −*π*/2 or *π*/2. When the phase difference between *t*_*xx*_ and *t*_*xy*_ is −*π*/2, only LCP incidence can be transmitted without polarization conversion. Likewise, when the phase difference between *t*_*xx*_ and *t*_*xy*_ is *π*/2, only RCP incidence can be transmitted without polarization conversion.

Hence, spin-selective transmission can be obtained when the above-mentioned condition is satisfied. Furthermore, the chirality of structures with *C*_4_-symmetry relative to the *z* axis but with no additional reflection symmetry can be engineered by designing the complex coefficients *t*_*xx*_ and *t*_*xy*_ to be reasonable.

## Results and Discussion

**Figure 1:**
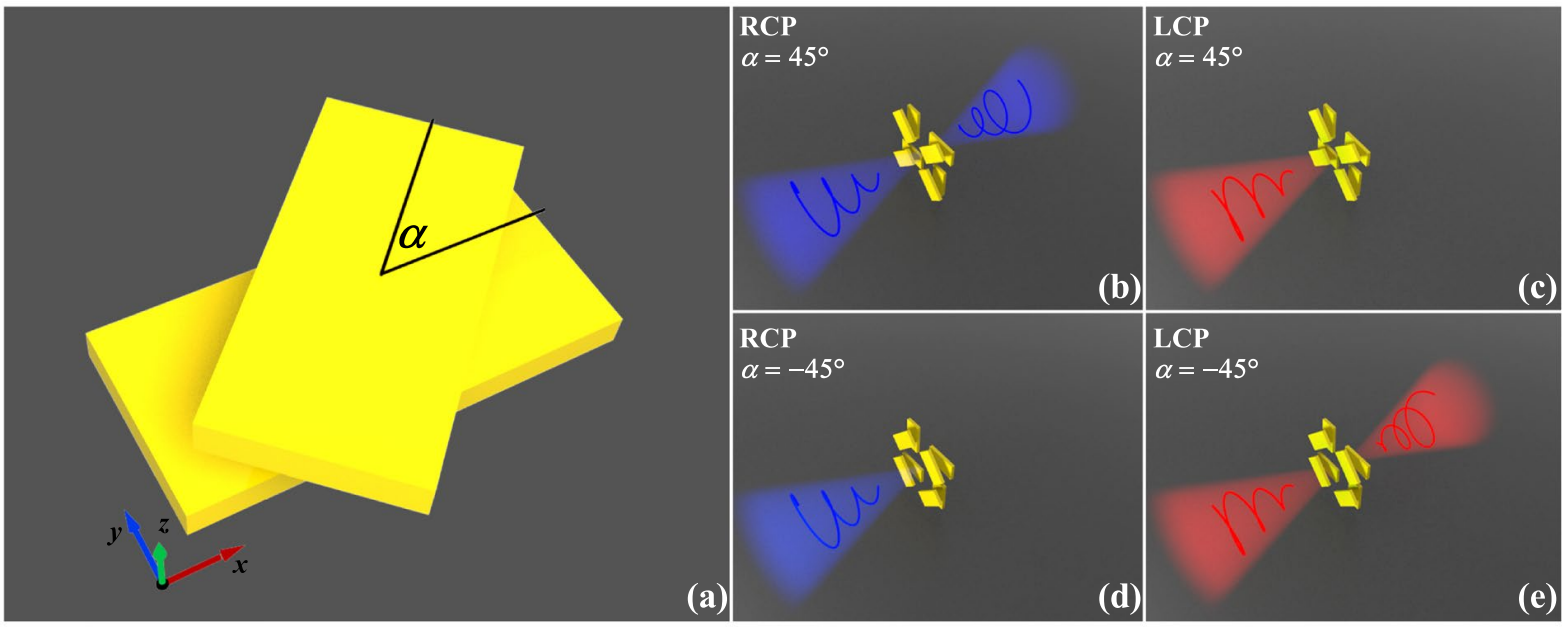
Design schematic for two-layer metasurfaces based on twisted nanorods. (**a**) The schematic of a twisted nanorod structure with orientation angle *α*. The designed two-layer metasurfaces, which are *C*_4_-symmetric, consist of four rotationally twisted nanorod structures with the same orientation angle. (**b**) to (**e**) Illustrations of spin-selective transmission in the two-layer metasurfaces for twisted nanorods with orientation angle *α* equal to 45° or −45°. The chiral optical responses exhibited by these two metasurfaces are opposite; thus, they can be treated as enantiomers.

We used the twisted gold nanorod structure shown in Fig. 1a to satisfy the above-mentioned conditions and achieve spin-selective transmission. The nanorods had length *l* of 300 nm, width *w* of 120 nm, and thickness *t* of 30 nm. The layer-to-layer separation distance *d* was 40 nm. The orientation angle *α* indicated the relative rotation angle between the top and bottom nanorods. The designed two-layer metasurfaces consisted of four rotationally twisted nanorod structures with the same orientation angle. Each designed metasurface had a unit-cell period *P* of 700 nm in both the *x* and *y* directions. The proposed metasurfaces were embedded in a SiO_2_ substrate to prevent wave interference at the interface of the air and substrate. The thickness *d*_2_ of the SiO_2_ covering on the top nanorods was 270 nm. This design can be fabricated on a SiO_2_ substrate with a mature layer-by-layer fabrication process^12, 23^. The designed metasurfaces display *C*_4_-symmetry relative to the *z* axis but with no additional reflection symmetry when the orientation angle *α* was not either 0° or 90°. Figure 1b,d show two different designs with orientation angle *α* equal to 45° and −45°, respectively.

**Figure 2:**
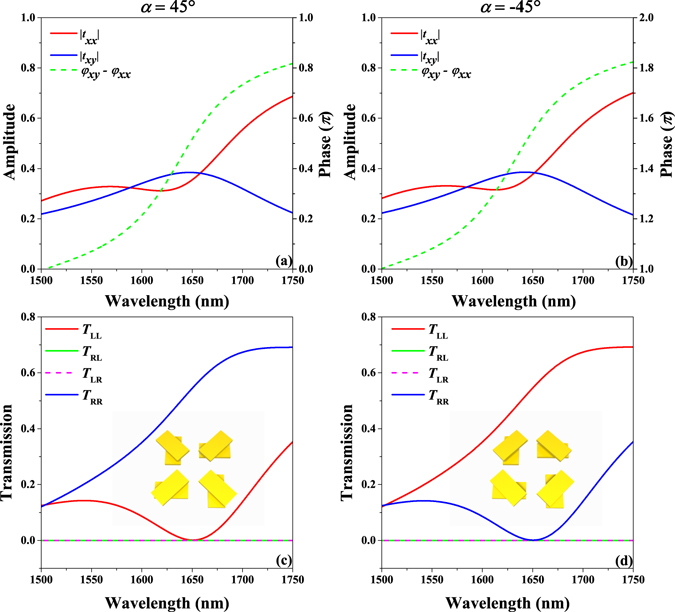
The analysis of **T** matrix coefficients of the designed metasurface. (**a**) and (**b**) Simulation results of the amplitudes and phase differences of linear base coefficients *t*_*xx*_ and *t*_*xy*_ of the two-layer metasurface with values of the orientation angle *α* of 45° and −45°, respectively. (**c**) and (**d**) Simulation results for the squared moduli *T*_*ij*_ = |*t*_*ij*_|^2^ of the circular base coefficients of the two-layer metasurface with values of the orientation angle *α* of 45° and −45°, respectively.

First, we simulate and discuss the **T** matrix coefficients of the designed metasurface with spin-selective transmission. Figure 2a,b show the amplitude and phase differences of linear base coefficients *t*_*xx*_ and *t*_*xy*_ for the two designed metasurfaces with orientation angle *α* of 45° and −45°, respectively. The metasurface with orientation angle *α* of 45° shows a phase difference between coefficients *t*_*xx*_ and *t*_*xy*_ close to *π*/2 around 1650 nm while the amplitudes of *t*_*xx*_ and *t*_*xy*_ are nearly equal. The metasurface with orientation angle *α* of −45° displays a phase difference between coefficients *t*_*xx*_ and *t*_*xy*_ close to −*π*/2 around 1650 nm while the amplitudes of *t*_*xx*_ and *t*_*xy*_ are nearly equal. The amplitude and phase difference of *t*_*xx*_ and *t*_*xy*_ of the proposed metasurfaces with orientation angles *α* of 45° and −45°, respectively, approximately fit the abovementioned conditions, which indicates the existence of spin-selective transmission in these metasurfaces around 1650 nm. The squared moduli *T*_*ij*_ = |*t*_*ij*_|^2^ of the circular base coefficients are simulated and shown in Fig. 2c,d for the designed metasurfaces with orientation angles *α* of 45° and −45°, respectively. The results show that for the metasurface with an orientation angle *α* of 45°, only *T*_RR_ does not equal to zero at 1650 nm, which means that only an RCP incident wave can be transmitted. In contrast, for the metasurface with an orientation angle *α* of −45°, only an LCP incident wave can be transmitted at 1650 nm, as shown in Fig. 1b to e. The transmission efficiency is over 50% around 1650 nm. Without SiO_2_ covering layer, the working wavelength of the spin-selective transmission will blue shift and its performance will reduce.

**Figure 3:**
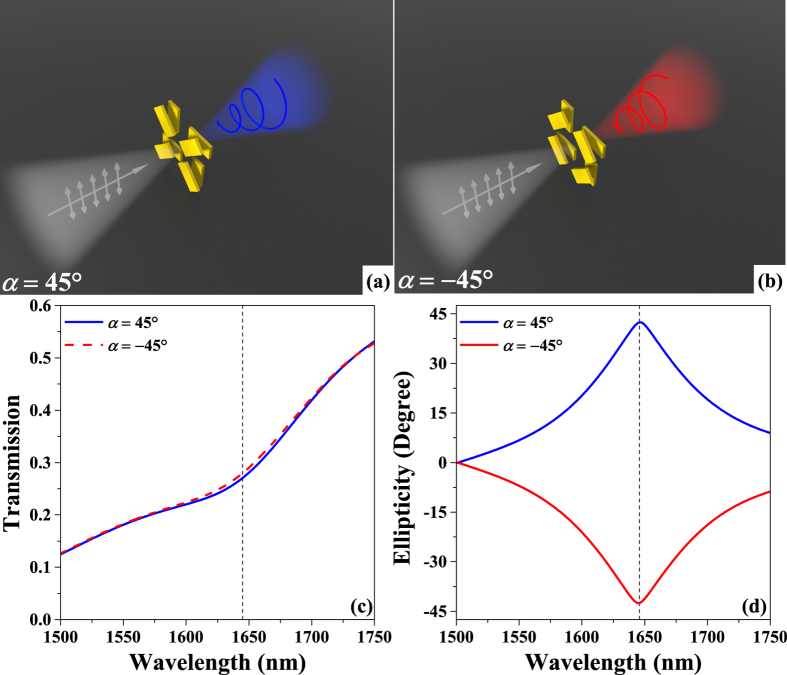
The performance of the designed metasurface as a spin selector. (**a**) and (**b**) Illustrations of the spin selection of the proposed two-layer metasurface with values of the orientation angle *α* of 45° and −45°, respectively. (**c**) and (**d**) Simulation results for transmission intensity and ellipticity when the incidence is *y*-polarized.

The spin-selective transmission in the designed metasurface is attributed to its chirality without any polarization conversion effect. Thus, these metasurfaces can be used for spin selection. The performance of the designed metasurface as a spin selector is shown in Fig. 3. Here, Fig. 3a,b provide illustrations of the spin selection in the designed metasurfaces. Arbitrarily polarized incident waves can be decomposed into LCP and RCP components. Only the LCP or RCP components of such incident waves can be transmitted in the designed metasurfaces with orientation angles *α* of −45° or 45°, respectively. When incidence is *y*-polarized, the transmission intensities are nearly equal across the examined waveband from 1500 nm to 1750 nm for those two metasurfaces. At the same time, their ellipticities have the same amplitudes but with opposite signs, as shown in Fig. 3c,d. The ellipticity of waves is defined as $\varphi =\frac{1}{2}\arcsin (\frac{2r}{1+{r}^{2}}\sin {\rm{\Delta }}\phi )$, where *r* represents the amplitude ratio given by *r* = |*t*_*y*_|/|*t*_*x*_| and Δ*φ* represents the phase difference between *t*_*x*_ and *t*_*y*_, given by Δ*φ* = *φ*_*y*_ − *φ*_*x*_, thus indicating the shape of the polarization ellipse. The ellipticity of the transmitted wave equals zero, meaning that the transmitted wave is ideally linearly polarized. When the ellipticity of the transmitted wave equals −45° or 45°, it represents LCP and RCP, respectively. The ellipticity of the transmitted wave is less than −40° from 1640 nm to 1652 nm and reaches a minimum value of −42.4° at 1646 nm in the designed metasurface with an orientation angle *α* of −45°. Also, the ellipticity of the transmitted wave exceeds 40° from 1640 nm to 1652 nm and reaches a maximum value of 42.4° at 1646 nm in the designed metasurface with an orientation angle *α* of 45°. The transmission intensity is around 28% at 1646 nm. For linear-polarized waves with arbitrary polarization directions, the amplitudes of the LCP and RCP components are in equal. Thus, the LCP and RCP components of such waves can be selected effectively by these two kinds of metasurfaces with equivalent transmission intensities around 28%, relative to the incident intensities, for wavelengths around 1646 nm.

**Figure 4:**
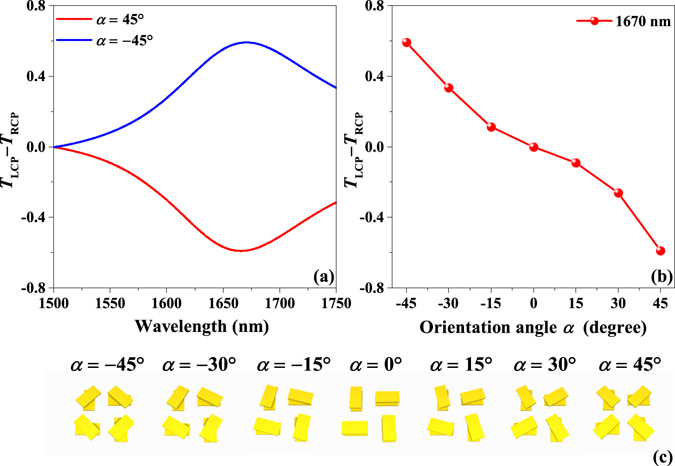
Characterizations of the chirality of the designed metasurfaces. (**a**) Simulation results for transmission difference Δ*T* = *T*_LCP_ −*T*_RCP_ of the two-layer metasurface with values of the orientation angle *α* of 45° and −45°, respectively. (**b**) Engineering of the transmission difference by orientation angle. (**c**) Schematic diagrams of various two-layer metasurfaces with different orientation angles in 15° increments from −45° to 45°.

Characterizations of the chirality of the designed metasurfaces can be obtained by simulating the transmission differences Δ*T* = *T*_LCP_ − *T*_RCP_, as Fig. 4 shows. Figure 4a depicts the transmission differences of the designed metasurfaces with orientation angles *α* of 45° and −45°, respectively. The amplitudes of the transmission difference exhibited by these two kinds of metasurfaces are equivalent; both reach a maximum value 0.58 at 1670 nm while their signs are opposite, which indicates their chiral optical responses are opposite. Thus, these two kinds of metasurfaces can be treated as enantiomers. Figure 4b shows the relationship between the transmission difference and orientation angle at 1670 nm, which indicates that the chirality of the metasurfaces can be engineered simply by changing the orientation of the twisted nanorods. The symmetry of the designed metasurface changes from *C*_4_-symmetry relative to the *z* axis, but with no additional reflection symmetry, to mirror symmetry perpendicular to the *z* axis as the orientation angle *α* varies from −45° to 0°. In addition, the symmetry of the designed metasurface changes from the mirror symmetry perpendicular to the *z* axis to a *C*_4_-symmetry relative to the *z* axis, but with no additional reflection symmetry, as the orientation angle *α* varies from 0° to 45°, as shown in Fig. 4c. These results indicate that the variation of chirality is consistent with the variation of the symmetry of the metasurface.

**Figure 5:**
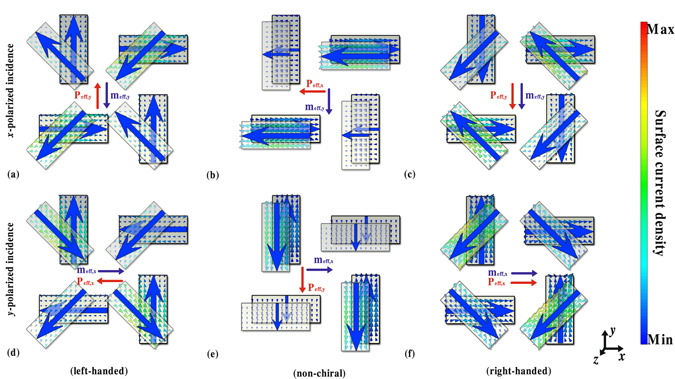
Simulated surface current distributions of the two-layer metasurfaces at 1670 nm. The surface current density for designed two-layer metasurfaces with orientation angle equal to (**a**) (**d**) 45°, (**b**) (**e**) 0° and (**c**) (**f**) −45°, respectively with (**a**–**c**) *x*-polarized and (**d–f**) *y*-polarized incidence. The induced electric and magnetic dipoles have antiparallel components and parallel components for orientation angle equal to 45° and −45° respectively, resulting in the opposite handedness of the two-layer metasurfaces.

In our simulations, we found that the near field interference in the designed two-layer metasurfaces plays an important role for the origin and the variation of its chirality. Thus, even though the designed two-layer metasurfaces consisted of four rotationally twisted nanorod structures, the chiral response of the designed two-layer metasurfaces cannot be directly obtained from the single twisted nanorod structure by using the advanced Jones calculus^24^. To further show the origin and the variation of the chirality in designed two-layer metasurfaces, we gave the dipolar analysis for the designed two-layer metasurfaces. Similar to natural chiral molecules, the specific combination of the electric and magnetic responses along the same direction, that is, parallel or antiparallel orientations with comparable magnitude, can induce strong chiral response^25, 26^. A right-handed enantiomer refers to the one whose components of effective electric and magnetic dipoles in the same direction are parallel orientation, while a left-handed enantiomer refers to the one whose components of effective electric and magnetic dipoles in the same direction are antiparallel orientation. To clarify the chiral response of the designed two-layer metasurfaces, we give the simulated surface current distribution at 1670 nm for three metasurfaces with different orientation angles for *x*- or *y*-polarized incidence in Fig. 5. It is noticeable that the direction of induced magnetic dipoles are always perpendicular to the polarization direction of the incidence and the direction of the effective electric dipoles of the top layer are always parallel to the polarization direction of the incidence. Moreover, the effective electric dipoles of the bottom layer have a component perpendicular to the polarization direction of the incidence while the orientation angle of the two-layer metasurfaces is not equal to 0°. For two-layer metasurfaces with orientation angle *α* of 45°, the components of its effective electric and magnetic dipoles perpendicular to the polarization direction of the incidence are antiparallel with each other, which result in a strong left-handed chiral response. There is no chiral response for two-layer metasurfaces with orientation angle *α* of 0° because its effective electric and magnetic dipoles have no component in the same direction. For two-layer metasurfaces with orientation angle *α* of −45°, the components of its effective electric and magnetic dipoles perpendicular to the polarization direction of the incidence are parallel with each other, which result in a strong right-handed chiral response. Moreover, with varying the orientation angle *α* from 45° to 0° or −45° to 0°, the magnitudes of the components of the effective electric and magnetic dipoles perpendicular to the polarization direction of the incidence will decrease and increase respectively, which result in the reduction of the chiral response.

**Figure 6:**
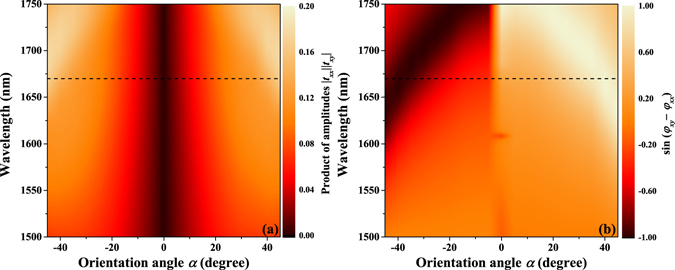
The relationship between the chiral response of the designed metasurface and its orientation angle. Simulation results for two-layer metasurfaces with different orientation angles and wavelengths. (**a**) Products of amplitudes |*t*_*xx*_|·|*t*_*xy*_| for the linear base transmission coefficients *t*_*xx*_ and *t*_*xy*_. (**b**) Sine value of phase difference *φ*_*xy*_− *φ*_*xx*_.

**Figure 7:**
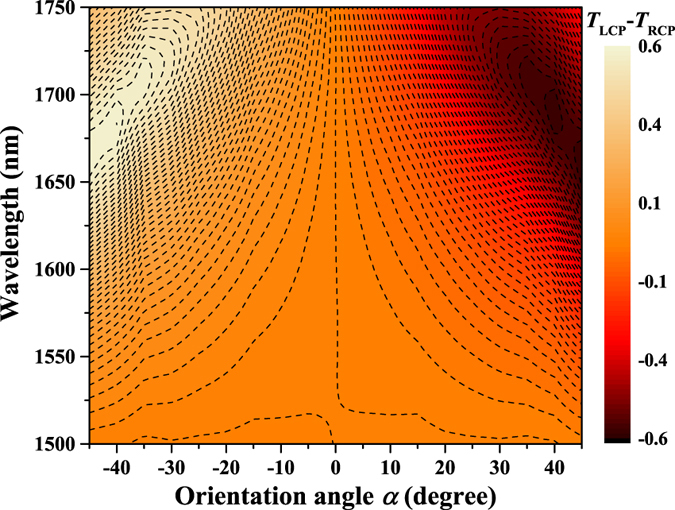
Simulation results for the transmission difference Δ*T* = *T*_LCP_−*T*_RCP_ of two-layer metasurfaces with different orientation angles and wavelengths. The black dashed lines indicate the boundaries of different values of Δ*T*.

The relationship between the variation of the chiral response of the designed metasurface and its orientation angle can be intuitively elucidated by examining the product of the amplitudes |*t*_*xx*_|·|*t*_*xy*_| and the sine value of the phase difference *φ*_*xy*_ − *φ*_*xx*_ of the linear-based transmission coefficients *t*_*xx*_ and *t*_*xy*_ of the designed metasurfaces with different orientation angles and wavelengths. Figure 6 illustrates a simulation of this relationship. Equation 6 indicates that the amplitude of the transmission difference is determined by the product of the amplitudes |*t*_*xx*_|·|*t*_*xy*_| and the sine value of the phase difference *φ*_*xy*_ − *φ*_*xx*_ while its sign is determined by the sign of the sine value of the phase difference. The black dashed lines in Fig. 6 indicate the products of the amplitudes and sine values of the phase differences for various orientation angles at 1670 nm. The product of the amplitudes and the amplitudes of the sine value decrease at first and then increase as the orientation angle *α* varies from −45° to 45°. Concurrently, the sign of the sine value changes from negative to positive. It is important to note that the variations of the product of amplitudes and the amplitudes of the sine values are symmetric as the orientation angle *α* varies from 0° to −45° and 0° to 45°. These results indicate that both the amplitude and the sign of the transmission difference are devisable by simply changing the orientation angle. Thus, the chiral optical response of the designed metasurface is devisable. Moreover, this relationship exists not only at 1670 nm but also across a broad waveband, especially between 1600 nm and 1750 nm. This finding indicates that conditions for chirality engineering exist in a broad waveband. The simulation results for transmission differences of the designed metasurfaces with different orientation angles and wavelengths are shown in Fig. 7. These results confirm our prediction. Therefore, the transmission difference of the designed metasurface can be varied continuously by changing the orientation angle across a broad waveband.

## Conclusion

We proposed the underlying theory, design specifications, and simulated demonstration of spin-selective transmission and chirality engineering in two-layer metasurfaces based on twisted nanorods in the near-infrared region. Using the results of the theoretical analysis employing advanced Jones calculus, we designed metasurfaces displaying *C*_4_-symmetry relative to the wave transmission direction but with no additional reflection symmetry. This designed metasurfaces then were optimized and used to simulate spin-selective transmission and chirality engineering. The simulation results are consistent with the theoretical prediction. Two-layer metasurfaces with opposite spin-selective transmission were realized at 1650 nm with over 50% efficiency. Thus, these two two-layer metasurfaces can be treated as enantiomers. The spin-selective transmission in the designed metasurfaces is attributed to their chirality with no polarization conversion effect. Thus, these metasurfaces can be used for spin selection between 1640 nm and 1652 nm with amplitudes of ellipticity greater than 40°. Moreover, we demonstrated that the chirality of the proposed metasurfaces can be engineered easily by simply changing the orientation angle of the twisted nanorods across a broad waveband. These results offer simple and straightforward rules for chirality engineering of metasurfaces and provide helpful insights and intriguing possibilities for applications in spin optics and enhanced chiral sensing in biological and chemical applications.

## Methods

Numerical simulations using the finite-differential time-domain (FDTD) method have been conducted to analyze the characterizations of the designed metasurface. Perfectly matched layers were used at the top and bottom of the simulation domain to completely absorb waves, leaving the simulation domain in the direction of propagation. Periodic boundary conditions were used in the *x* and *y* directions representing a periodical structure. The permittivity of the SiO_2_ was taken as 2.25. The dispersion function of gold was defined by the Drude model with a plasma frequency *ω*_*p*_ of 1.37 × 10^16^ s^−1^ and damping constant γ of 1.224 × 10^14^ s^−1^ ^27, 28^.
